# Encryption Algorithm of Multiple-Image Using Mixed Image Elements and Two Dimensional Chaotic Economic Map

**DOI:** 10.3390/e20100801

**Published:** 2018-10-18

**Authors:** A. A. Karawia

**Affiliations:** 1 Department of Mathematics, Faculty of Science, Mansoura University, Mansoura 35516, Egypt; abibka@mans.edu.eg or; 2 Computer Science Unit, Deanship of Educational Services, Qassim University, P.O. Box 6595, Buraidah 51452, Saudi Arabia

**Keywords:** image encryption, multiple-image encryption, two-dimensional chaotic economic map, security analysis, 68U10, 68P25, 94A60

## Abstract

To enhance the encryption proficiency and encourage the protected transmission of multiple images, the current work introduces an encryption algorithm for multiple images using the combination of mixed image elements (MIES) and a two-dimensional economic map. Firstly, the original images are grouped into one big image that is split into many pure image elements (PIES); secondly, the logistic map is used to shuffle the PIES; thirdly, it is confused with the sequence produced by the two-dimensional economic map to get MIES; finally, the MIES are gathered into a big encrypted image that is split into many images of the same size as the original images. The proposed algorithm includes a huge number key size space, and this makes the algorithm secure against hackers. Even more, the encryption results obtained by the proposed algorithm outperform existing algorithms in the literature. A comparison between the proposed algorithm and similar algorithms is made. The analysis of the experimental results and the proposed algorithm shows that the proposed algorithm is efficient and secure.

## 1. Introduction

A huge number of images are produced in many fields, such as weather forecasting, military, engineering, medicine, science and personal affairs. Therefore, with the fast improvement of computer devices and the Internet, media security turns into a challenge, both for industry and academic research. Image transmission security is our target. Many authors have proposed many single-image encryption algorithms to solve this problem [1,2,3,4,5,6,7,8]. Single-image encryption algorithms involve those using a chaotic economic map [1,2], using a chaotic system [3], via one-time pads-a chaotic approach [4], via pixel shuffling and random key stream [5], using chaotic maps and DNA encoding [6] and using the total chaotic shuffling scheme [7]. In [8], the authors proposed two secret sharing approaches for 3D models using the Blakely and Thien and Lin schemes. Those approaches reduce share sizes and remove redundancies and patterns, which may ease image encryption. The authors in [9] concluded that the dynamic rounds chaotic block cipher can guarantee the security of information transmission and realize a lightweight cryptographic algorithm. A single-image can encrypt multiple images repeatedly, but the efficiency of that encryption is always unfavorable. Researchers have increased their attention towards multiple-image encryption because a high efficiency of secret information transmission is required for modern multimedia security technology. Many multiple-image algorithms have been presented. The authors of [10] presented a multiple-image algorithm via mixed image elements and chaos. A multiple-image algorithm using the pixel exchange operation and vector decomposition was proposed in [11]. In [12], the authors presented an algorithm using mixed permutation and image elements. The authors presented multiple-image encryption via computational ghost imaging in [13]. In [14], the authors proposed an algorithm using an optical asymmetric key cryptosystem. A multiple-image encryption algorithm based on spectral cropping and spatial multiplexing was presented in [15]. The authors of [16] proposed a multiple-image encryption algorithm based on the lifting wavelet transform and the XOR operation based on compressive ghost imaging scheme. Even with this large number of proposed algorithms, some practical problems still exist. For instance, some multiple-image algorithms have faced the problem that the original images cannot be recovered completely [17,18,19]. Those algorithms were used to encrypt multiple images, but the corresponding original images were not recovered completely. This leads to lossy algorithms, which are not appropriate for those applications needing images with high visual quality. Another problem is that the complex computations of some algorithms affect the encryption efficiency [20,21]. Therefore, good techniques are required for solving these problems [22]. In the current paper, a new efficient multiple-image encryption algorithm using mixed image elements (MIES) and a two-dimensional chaotic economic map is proposed. The advantages of this algorithm are that it is able to recover plain images completely and simplifies the computations. Experimental results demonstrate its practicality and high proficiency.

The rest of the paper is organized as follows. The pure image elements (PIES) and the MIES are defined in Section 2. In Section 3, a brief introduction to the two-dimensional chaotic economic map is presented. The secret key generation is presented in Section 4. In Section 5, a new encryption algorithm of multiple images is designed. Experimental results and analyses are introduced in Section 6. Section 7 presents a comparison between the proposed algorithm and the identical algorithms. Conclusions are given in Section 8.

## 2. PIES and MIES

**Figure 1:**
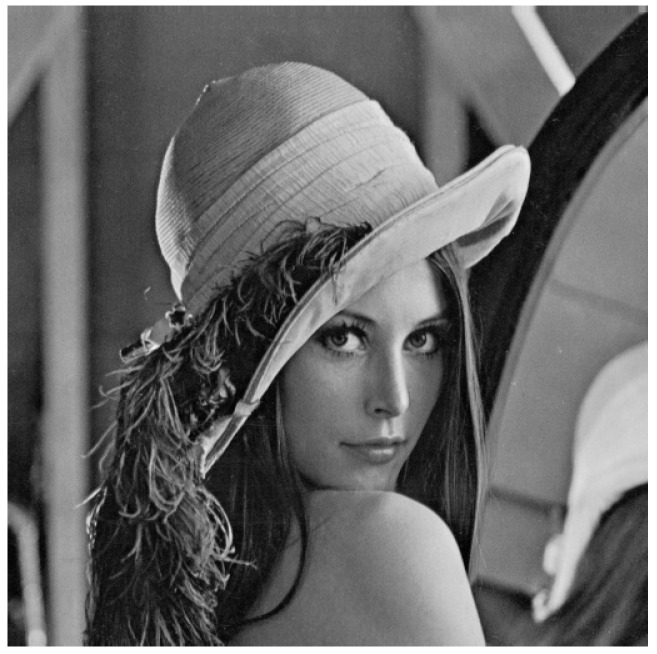
Lena image with a 512×512 size.

**Figure 2:**
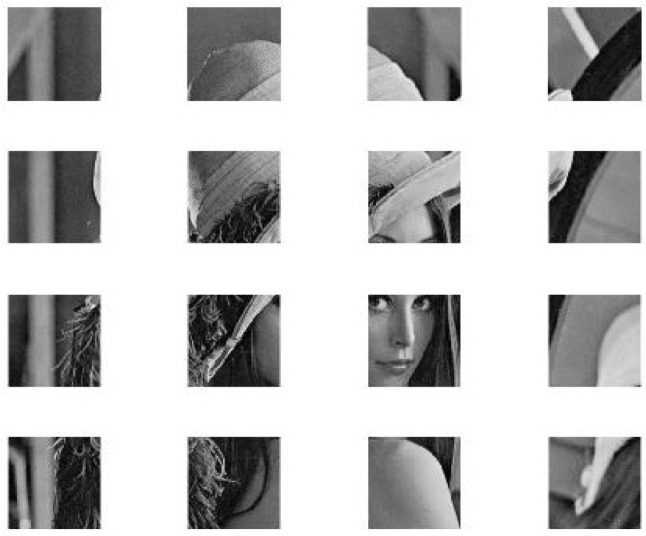
Pure image elements (PIES) of the Lena image with a 512×512 size.

Matrix theory can be used to divide a big matrix into many small matrices and vice versa. Furthermore, in the image processing field, it is simple to divide an image into many small images and vice versa. For instance, Figure 1 can be divided into 16 small images with an equal size, as displayed in Figure 2. Therefore, the original image can be retrieved from these 16 images.

Assume that $O1_{m\times n},O2_{m\times n},\cdots ,Ok_{m\times n}$ are *k* original images. $O1_{m\times n}$ can be divided into a small images set, $\left\{B1_{i}\right\}$. Each element $B1_{i}\in \left\{B1_{i}\right\}$ is referred to as the pure image element. On the other hand, *k* sets of PIES $\left\{B1_{i}\right\}$, $\left\{B2_{i}\right\}$, ⋯, $\left\{Bk_{i}\right\}$ can be created, which correspond to $O1_{m\times n},O2_{m\times n},\cdots ,Ok_{m\times n}$, respectively. A large set $C=\left\{B1_{i}\right\}\cup \left\{B2_{i}\right\}\cup \cdots \cup \left\{Bk_{i}\right\}$ can be obtained by mixing all PIES together. Each element $C_{i}\in C$ is referred to as the mixed image element.

The current paper presents a new encryption algorithm of multiple images using MIES and the two-dimensional chaotic economic map. The secret key is very important to restore the original images from the MIES.

## 3. The Two-Dimensional Chaotic Economic Map

The study of the following two-dimensional chaotic economic system (dynamical system) was introduced in [23]:

(1) $$\left.\begin{array}{cc} \alpha_{n+1} & =\alpha_{n}+k\left[a-c-\frac{b\alpha_{n}}{\gamma_{n}}-b\log\left(\gamma_{n}\right)\right],\\ \beta_{n+1} & =\beta_{n}+k\left[a-c-\frac{b\beta_{n}}{\gamma_{n}}-b\log\left(\gamma_{n}\right)\right], \end{array}\right\}$$

where:

$$\gamma_{n}=\alpha_{n}+\beta_{n},\;n=0,1,2,\ldots$$

**Figure 3:**
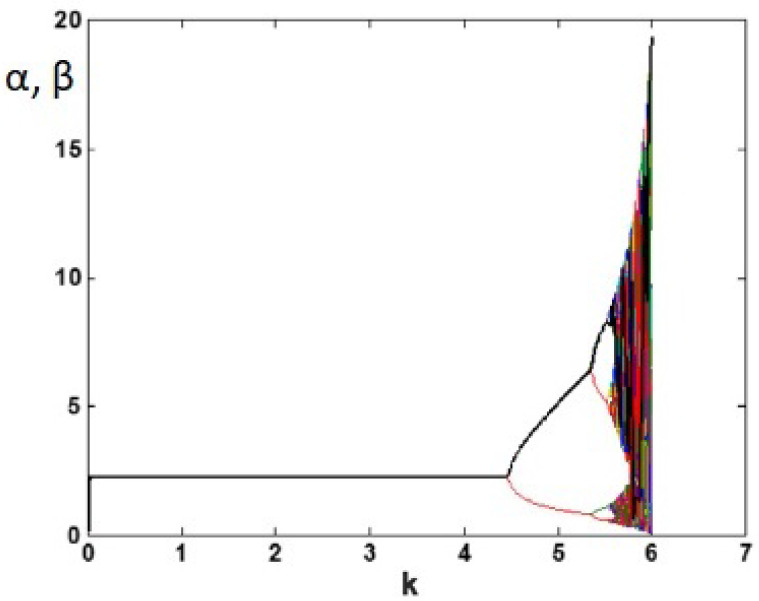
The chaotic behavior of the map (1) at $a=3,\;b=1,\;c=1,\;\alpha_{0}=0.19,\;\beta_{0}=0.15$ and k∈[0,6.0001].

**Figure 4:**
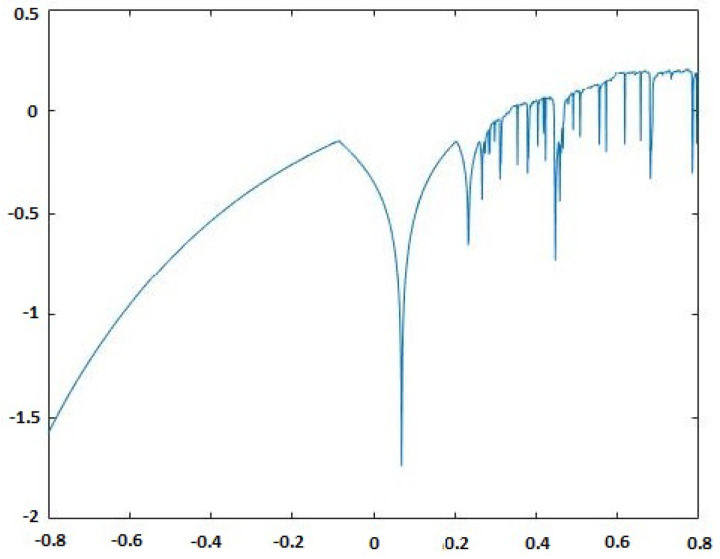
Lyapunov exponent for the chaotic economic map (1) at $a=3,\;b=1,\;c=1,\;\alpha_{0}=0.19,\beta_{0}=0.15$ and k∈[0,6.0001].

There are six parameters in the chaotic economic map (1). These parameters have economic significance; the parameter a>0 is used to capture the economic market size, while the market price slope is referred to by the parameter b>0. To obtain a chaotic region, *a* must be greater than *b* and *c*. A fixed marginal cost parameter is denoted by c≥0, and the speed of adjustment parameter k>0. The chaotic behavior of the chaotic economic map (1) at $a=3,b=1,c=1,\alpha_{0}=0.19,\beta_{0}=0.15$ and k∈[0,6.0001] is shown in Figure 3. In the current paper, the parameters a=3,b=1,c=1 and k=5.9 of the map (1) have been chosen in the chaotic region having positive Lyapunov exponents, as displayed in Figure 4.

## 4. The Secret Key Generation

Let $B=(b_{ij})$, i=1,2,…,M,j=1,2,…,N, be the big image created by combining the *k* original images of size m×, where $b_{ij}$ refers to the pixel value at the position (i,j) and (M,N) is the size of the big image B. The key mixing proportion factor can be used to calculate $K_{z},z=1,2,3,\cdots ,10,$ as follows:

(2) $$K_{z}=\frac{1}{256}mod\left(\sum_{i=\frac{(z-1)M}{8}+1}^{\frac{zM}{8}}\sum_{j=1}^{N}b_{ij},256\right)$$

Then, update the initial condition $\Theta_{0}$ using the following formula:

(3) $$\Theta_{0}\leftarrow \frac{\left(\Theta_{0}+K\right)}{2},$$

where $\Theta_{0}=x_{10},x_{20},x_{30},x_{40},r_{10},r_{20},r_{30},r_{40},q_{10},q_{20}$ and $K=K_{j},j=1,2,\cdots ,10$, receptively.

After that, take four initial values, $x_{10},x_{20},x_{30},x_{40}$, four parameters for the logistic map, $r_{10},r_{20},r_{30},r_{40}$, two initial values for the system, $q_{10},q_{20}$, and four system parameters, a,b,c,k.

## 5. The Proposed Multiple-Image Algorithm

**Figure 5:**
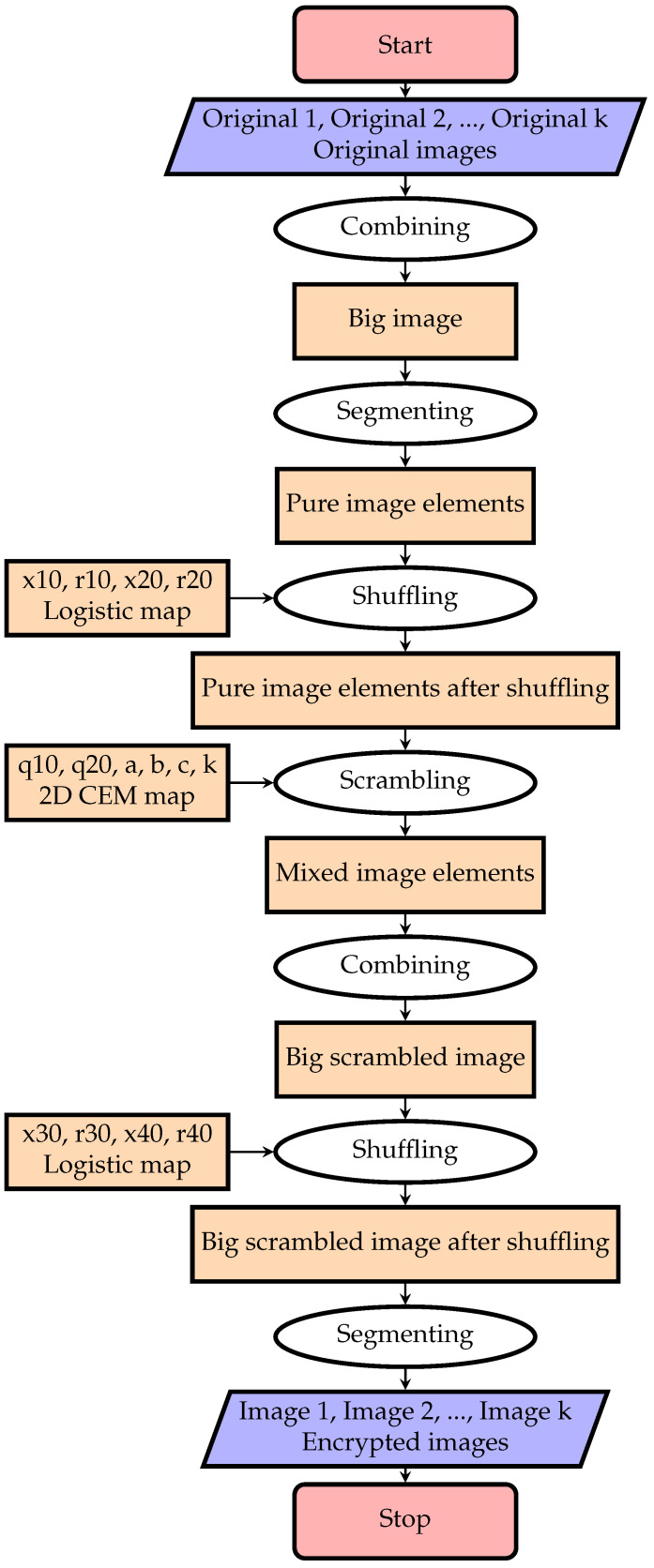
Flowchart of multiple-image encryption.

To encrypt multiple images jointly, the current work presents a new encryption algorithm of multiple images using MIES and the two-dimensional chaotic economic map. The flowchart of the new encryption algorithm is shown in Figure 5.

The proposed algorithm is processed as follows:

In the multiple-image decryption, the same chaotic economic sequences are generated on the multiple-image encryption that will be used to recover the original images and using the inverse steps of Algorithm 1.

**Algorithm 1 ** Multiple-image encryption
**Input:***k* original images, O1,O2,⋯,Ok, $x_{i0}$, $r_{i0},i=1,2,3,4$ for logistic shuffling and          $a,b,c,k,\alpha_{0},\beta_{0}$ for the two-dimensional chaotic economic map (1).**Output:** Encrypted images Image1,Image2,⋯,Imagek.**Step 1:** Create a big image by combining the *k* original images.**Step 2:** Divide the big image into PIES of $m_{1}\times n_{1}$ size such that $\mathrm{mod}(m,m_{1})=0$,            $\mathrm{mod}(n,n_{1})=0$ and the original images with size m×n.**Step 3:** Shuffle the pixels of PIES using the logistic map:            $x_{n}=rx_{n-1}\left(1-x_{n-1}\right),n=1,2,3,\cdots$, and use the parameters $\left(x_{10},r_{10}\right)$ and $\left(x_{20},r_{20}\right)$            for shuffling the rows and the columns, respectively.**Step 4:** Generate the chaotic economic sequences using:$$\alpha_{n+1}=\alpha_{n}+k\left[a-c-\frac{b\alpha_{n}}{\gamma_{n}}-b\log\left(\gamma_{n}\right)\right],$$
$$\beta_{n+1}=\beta_{n}+k\left[a-c-\frac{b\beta_{n}}{\gamma_{n}}-b\log\left(\gamma_{n}\right)\right],$$           where $n=0,1,2,\cdots ,a=3,b=1,c=1,\alpha_{0}=0.001,\beta_{0}=0.002$ and k=5.9.**Step 5:** Do the following preprocessing for the generated values in **Step 4**:            $\alpha_{i}=floor\left(mod\left(\alpha_{i}\times 10^{14},256\right)\right)$ and $\beta_{i}=floor\left(mod\left(\beta_{i}\times 10^{14},256\right)\right)$,**Step 6:** Convert $\alpha_{i}$ and $\beta_{i}$ into binary vectors, say *A* and *B*, respectively.**Step 7:** Perform a bit-wise XOR  between *A* and *B*, say *C* = bitxor(*A*,*B*).**Step 8:** Convert the pixels of shuffled PIES into a binary vector, say *D*.**Step 9:** Perform a bit-wise XOR between *C* and *D*, say *E* = bitxor(*C*,*D*).**Step 10:** Combine these mixed scrambled PIES into a big scrambled image.**Step 11:** Shuffle the pixels of the big scrambled image using the logistic map, and use the             parameters $\left(x_{30},r_{30}\right)$ and $\left(x_{40},r_{40}\right)$ for shuffling the rows and the columns,             respectively.**Step 12:** Divide it into images of equal size m×n. These images are viewed as encrypted              images, say Image1,Image2,⋯,Imagek.**Step 13:** End.


## 6. Experimental Results and Analyses

**Figure 6:**
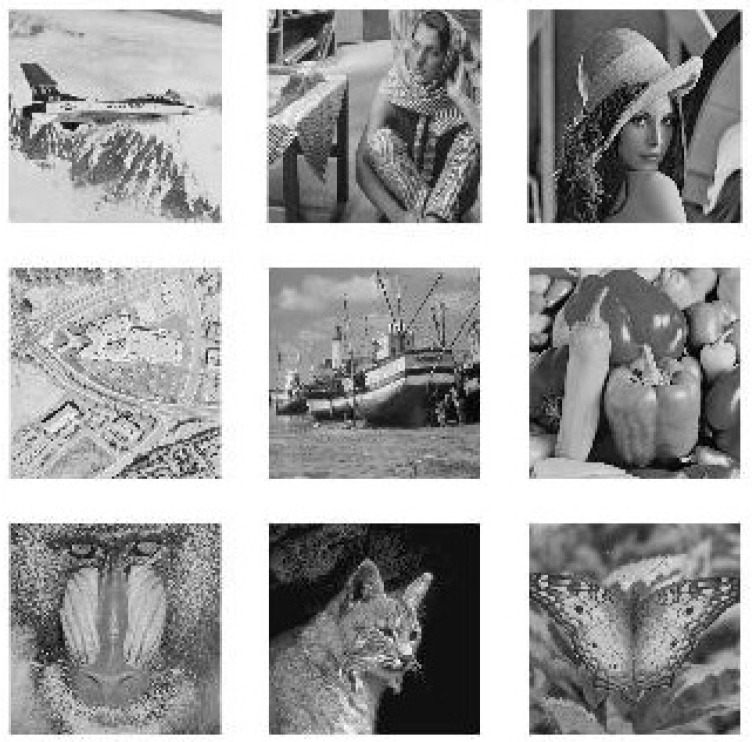
Original images.

**Figure 7:**
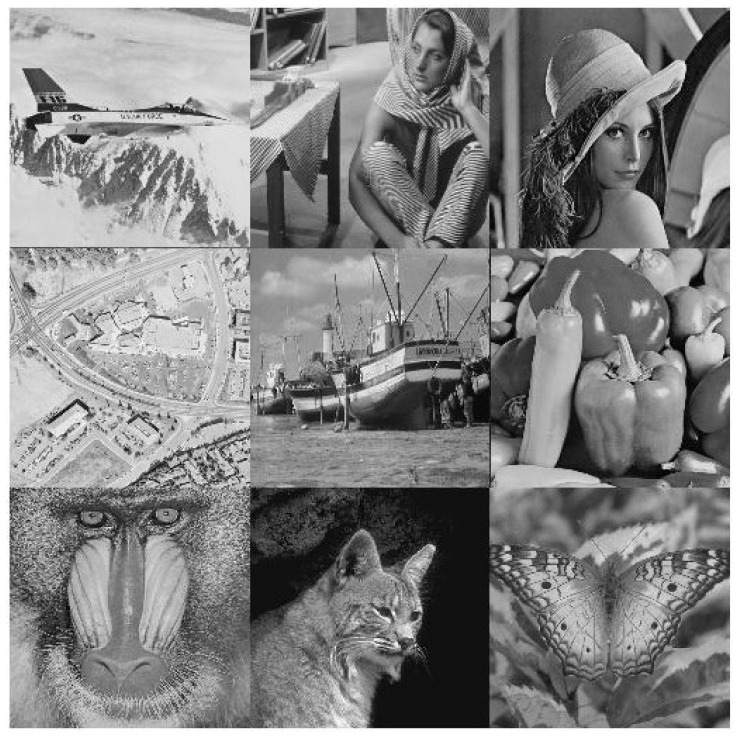
Big image.

**Figure 8:**
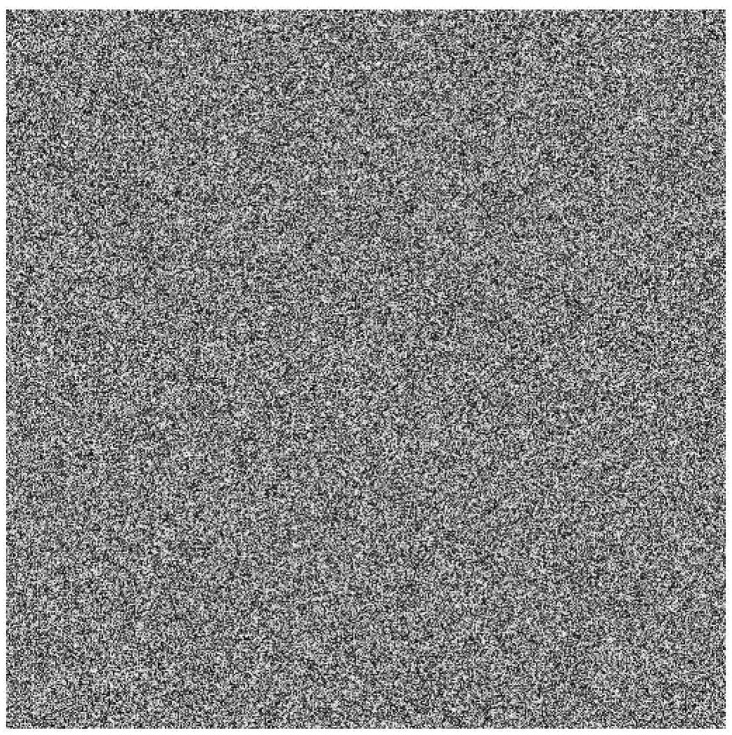
Mixed image elements (MIES) with equal size 4×4.

**Figure 9:**
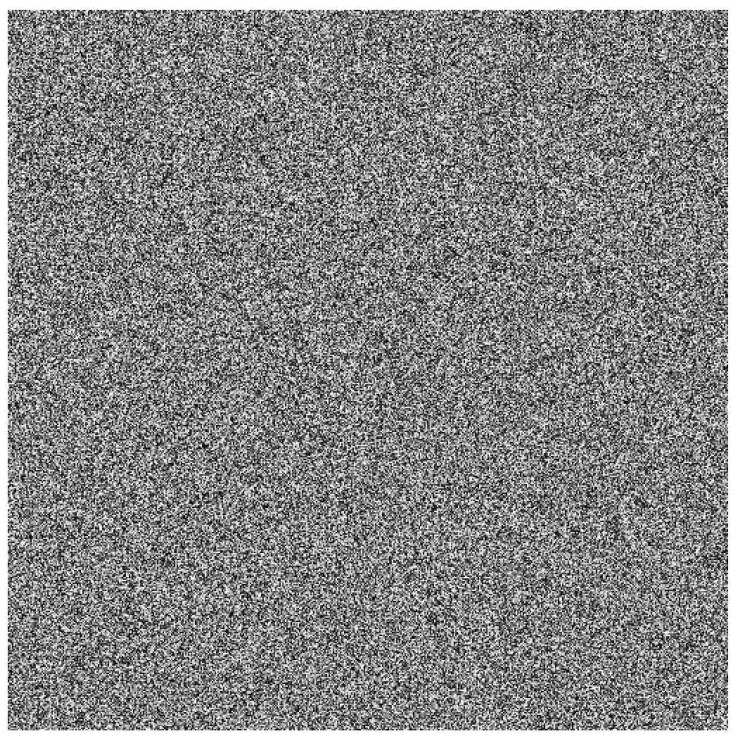
MIES with equal size 8×8.

**Figure 10:**
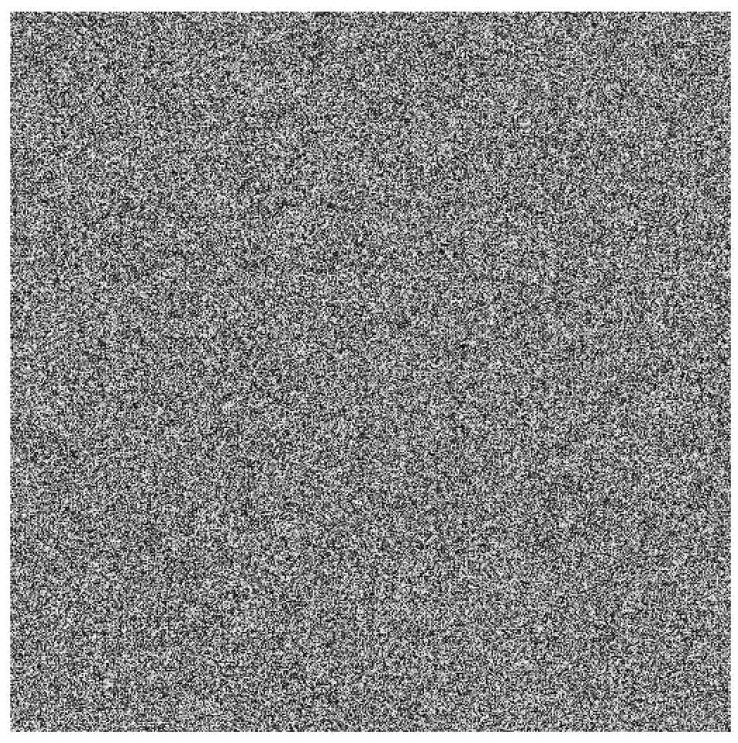
MIES with equal size 16×16.

**Figure 11:**
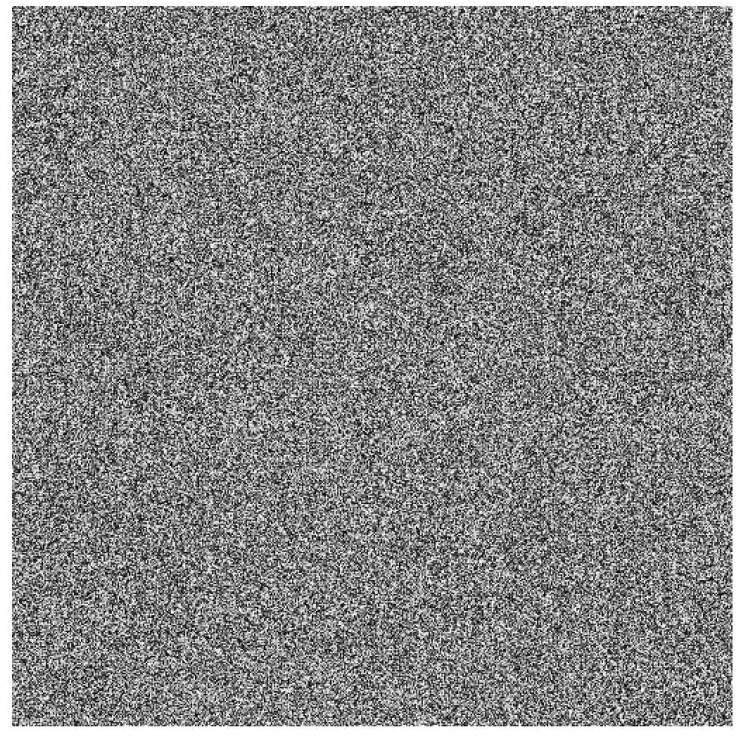
MIES with equal size 32×32.

**Figure 12:**
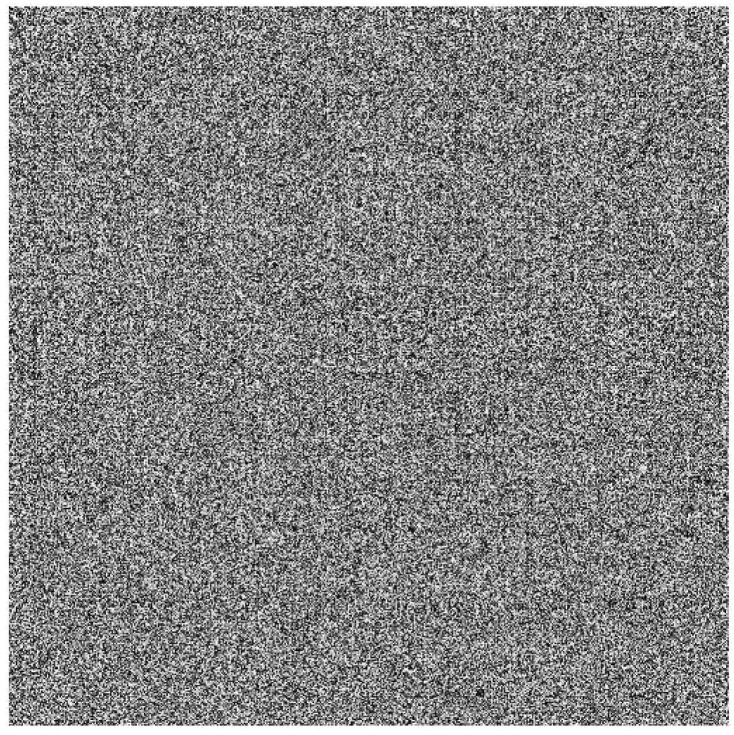
MIES with equal size 64×64.

**Figure 13:**
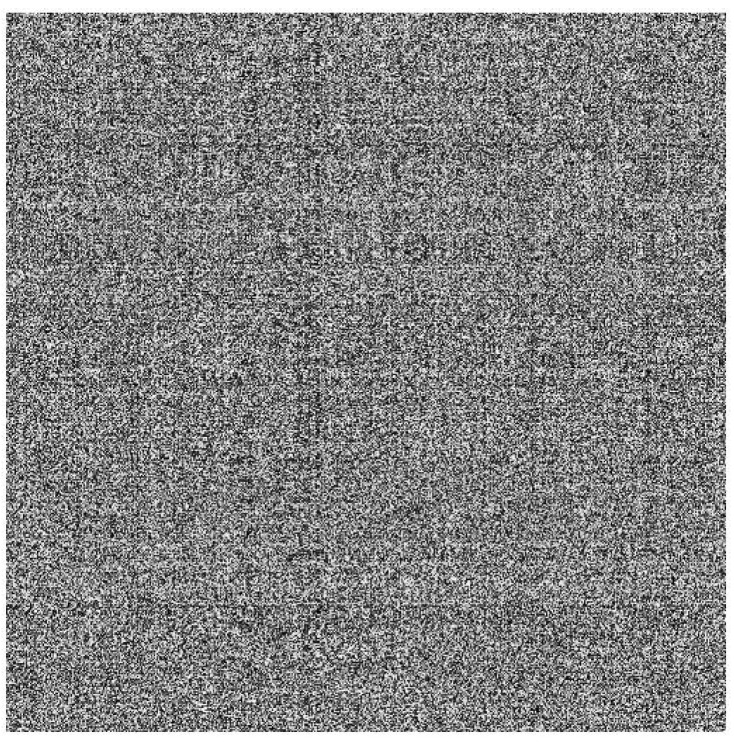
MIES with equal size 128×128.

**Figure 14:**
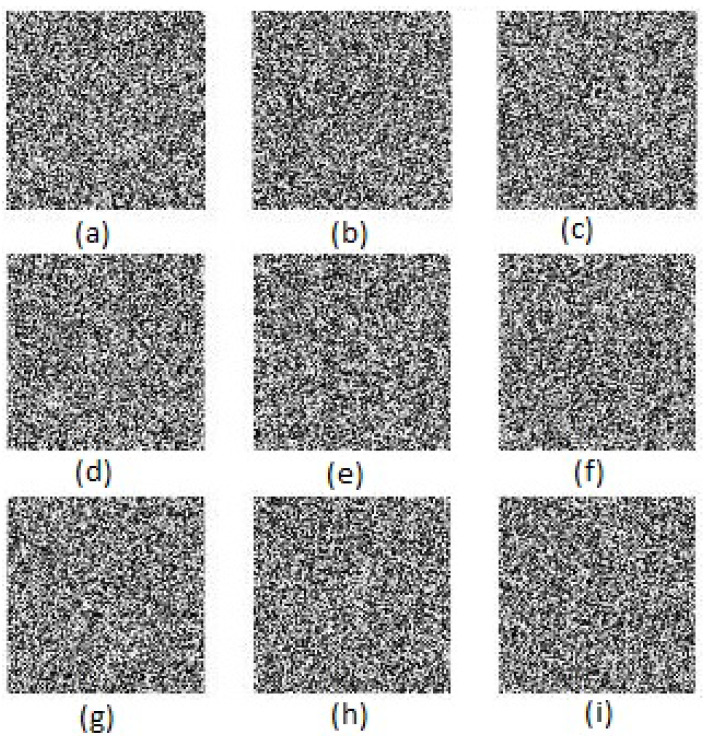
Encrypted images. (**a**) encrypted image of airplane. (**b**) encrypted image of barbara. (**c**) encrypted image of lena. (**d**) encrypted image of aerial. (**e**) encrypted image of boat. (**f**) encrypted image of peppers. (**g**) encrypted image of baboon. (**h**) encrypted image of cat. (**i**) encrypted image of butterfly.

**Figure 15:**
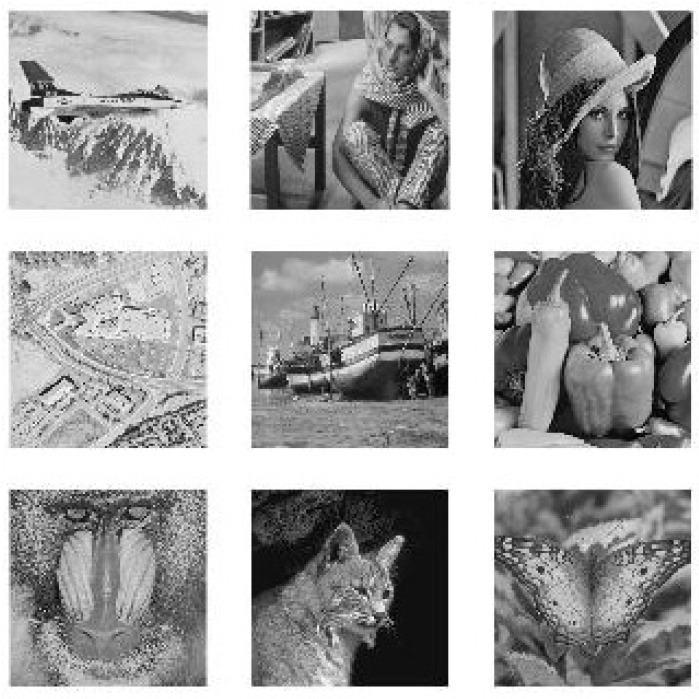
Decrypted images.

To show the efficiency and robustness of the proposed algorithm, nine (k=9) original gray images of a 512×512 size are shown in Figure 6. Let $x_{10}=0.1,x_{20}=0.2$ be the initial values and $r_{10}=3.9985,r_{20}=3.9988$ be the parameters of the logistic map for shuffling the PIES. Furthermore, let $x_{30}=0.3,r_{30}=3.9984$ and $x_{40}=0.4,r_{40}=3.9986$ be the initial values and the parameters of the logistic map for shuffling the big scrambled image. Let $\alpha_{0}=0.19,\beta_{0}=0.15,a=3,b=1,c=1$ and k=5.9 be the initial values and the control parameters of the chaotic economic map (1). All nine original gray images are combined into one big image, which is displayed in Figure 7. Figure 8, Figure 9, Figure 10, Figure 11, Figure 12 and Figure 13 show the big scrambled images that correspond to the MIES of equal sizes 4×4, 8×8, 16×16, 32×32, 64×64 and 128×128, respectively. The corresponding encrypted images of MIES with size 64×64 are shown in Figure 14. Furthermore, the corresponding decrypted images are displayed in Figure 15. Experiments are performed with MATLAB R2016a software to execute the proposed algorithm on a laptop with the following characteristics: 2.40 GHz Intel Core i7-4700MQ CPU and 12.0 GB RAM memory.

The performance of the presented multiple-image encryption algorithm is investigated in detail as follows.

### 6.1. Analysis of the Key Space

A large key space is required to make the brute-force attack infeasible [10]. In the proposed algorithm, the key space was selected as follows. In the logistic map, $x_{10},\;r_{10},\;x_{20},r_{20},\;x_{30},\;r_{30},\;x_{40},\;r_{40}$ were selected to shuffle rows and columns. $\alpha_{0},\beta_{0},a,b,c$ and *k* were selected for the chaotic economic map (1). Then, the key space size was $10^{15\times 14}=10^{210}$ if the computer precision were $10^{-15}$. Table 1 shows that the key spaces in [10,20,22] were less than the presented key space. Therefore, it was large enough to make the brute-force attack infeasible.

### 6.2. Analysis of the Key Sensitivity

**Figure 16:**
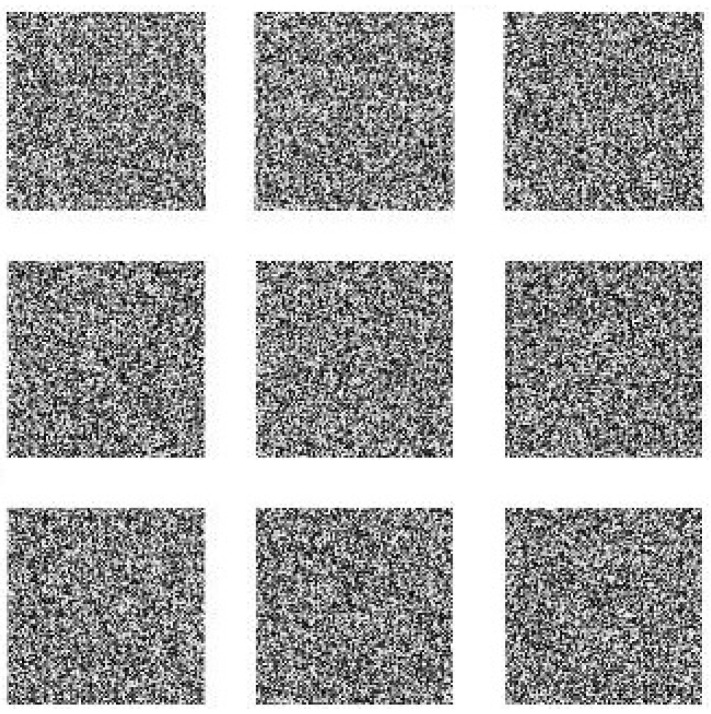
Decrypted images with the correct secret key, except $\alpha_{0}=0.190000000000001$, instead of $\alpha_{0}=0.19$.

An excellent multiple-image encryption algorithm should be very sensitive to modifying any key of the encryption and the decryption processes. Making a small modification to the key of the encryption, the output encrypted image (the second one) should be absolutely unlike the first encrypted image. Furthermore, if the encryption and decryption keys have a small difference, then the encrypted image cannot be restored correctly [23]. The restored images of the encrypted images in Figure 14 with a small change of the secret key, say $\alpha_{0}=0.190000000000001$ instead of $\alpha_{0}=0.19$, and the other parameters unchanged, are shown in Figure 16. The result shows that a small modification of the key can lead to completely different encrypted images, and the restoration of original images becomes very complicated. As the sensitivity of $x_{10},\;r_{10},\;x_{20},\;r_{20},\;x_{30},\;r_{30},\;x_{40},\;r_{40},\;\beta_{0},\;a,\;b,\;c$ and *k* was the same as $\alpha_{0}$, their examples are omitted here.

### 6.3. Analysis of the Histogram

**Figure 17:**
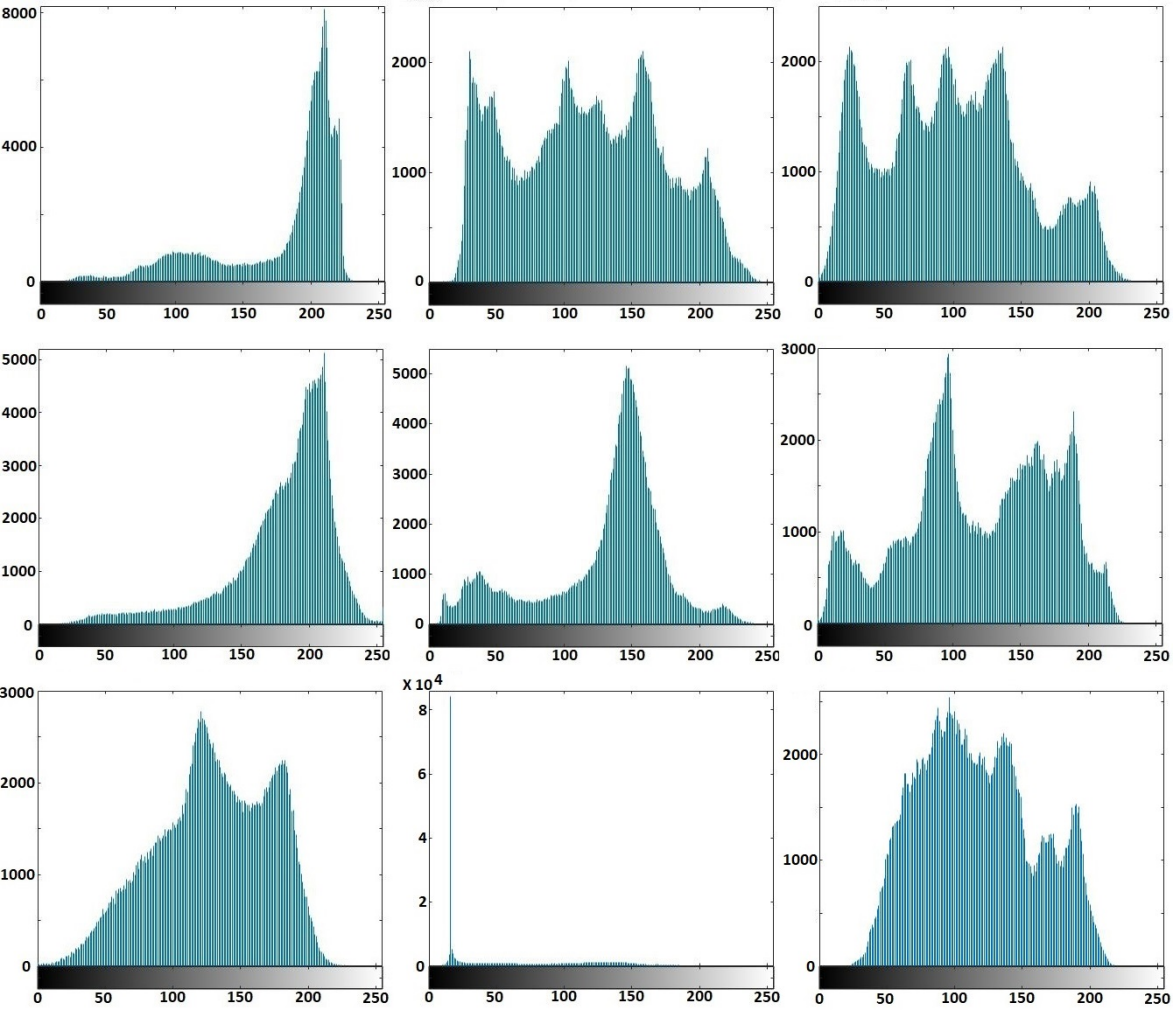
Histograms of the original images.

**Figure 18:**
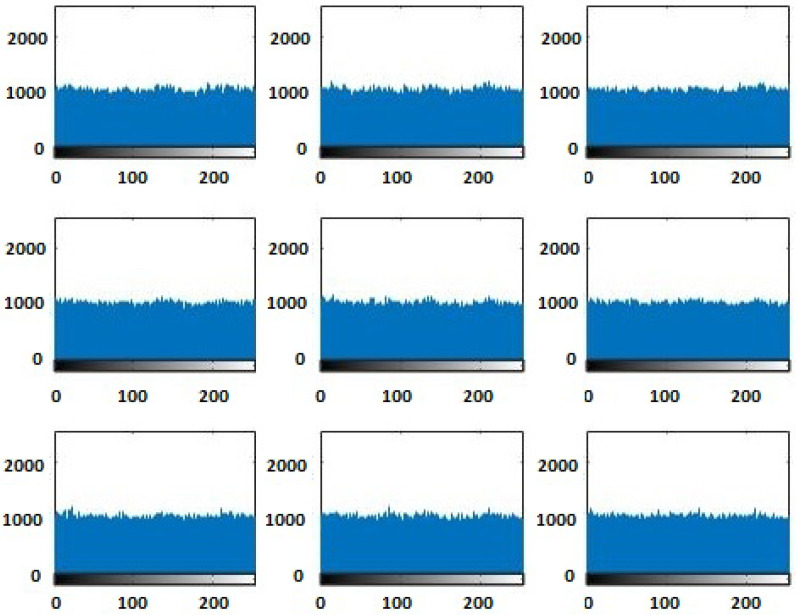
Histograms of the encrypted images.

The original images’ histograms are shown in Figure 17, while the corresponding encrypted images histograms are shown in Figure 18. Figure 16 and Figure 18 display that the original images had different histograms, while the corresponding encrypted images histograms had a uniform distribution approximately. Therefore, the encryption process damaged the original images’ features.

### 6.4. Analysis of Histogram Variance

The histogram variance of a gray image is defined by:

(4) $$Var\left(V\right)=\frac{1}{256}\sum_{i=0}^{255}{\left[v_{i}-E\left(V\right)\right]}^{2},$$

where $E\left(V\right)=\frac{1}{256}\sum_{i=0}^{255}v_{i}$ and *V* is the pixel number vector of 256 gray levels.

**Figure 19:**
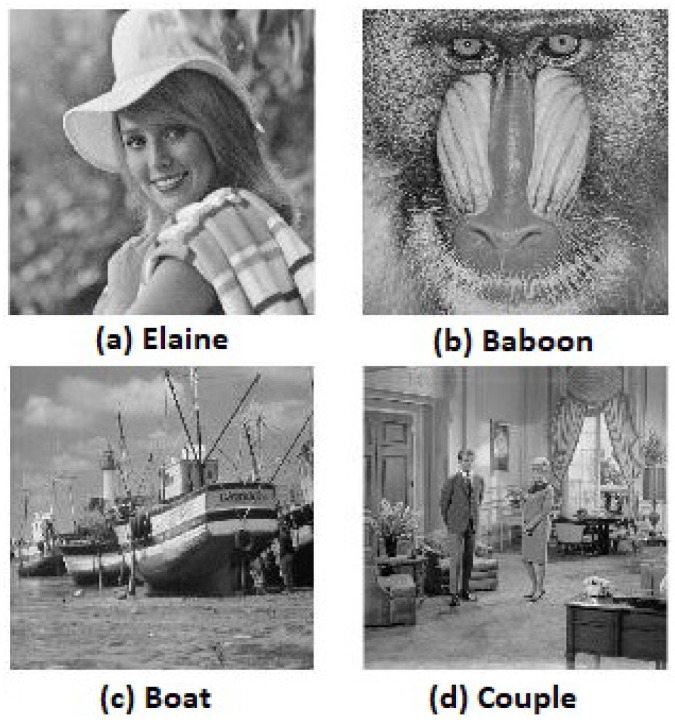
Input images. (**a**) Elaine; (**b**) Baboon; (**c**) Boat; (**d**) Couple.

This can clarify the impact of the encrypted image to some degree. In a perfect random image, all the gray levels have equal probabilities. Therefore, the histogram variance equals zero. Therefore, the histogram variance of the encrypted image via an effective encryption algorithm should tend to zero. Table 2 shows the values of the histogram variances of the encrypted images of the original images in Figure 19 via Tang’s algorithm [20], Zhang’s algorithm [10] and the proposed algorithm, respectively.

### 6.5. Analysis of Information Entropy

In a digital image, the information entropy can be an indicator of the pixel values’ distribution. For a perfect random image, $P\left(v_{i}\right)=\frac{1}{256},i=0,1,2,\cdots ,255$, where $v_{i}$ is the *i*-th gray level of the image and $P(v_{i})$ is the probability of $v_{i}$. Furthermore, it has information entropy =8. Now, the information entropy is computed by [24]:

(5) $$H\left(V\right)=-\sum_{i=0}^{255}P\left(v_{i}\right)log_{2}P\left(v_{i}\right)$$

Table 3 lists the values of information entropy for the encrypted images in Figure 14. The information entropy of the encrypted images of the proposed algorithm is better than the information entropy of the encrypted images of the multiple-image encryption algorithm in [10]. Therefore, the efficiency and security of the proposed algorithm is clear.

### 6.6. Analysis of the Correlation Coefficients

In the image encryption, the correlation coefficient was used to measure the correlation between two neighboring pixels, horizontally, vertically and diagonally neighboring. It is evaluated by [25]:

(6) $$R_{V_{1}V_{2}}=\frac{COV(V_{1},V_{2})}{\sqrt{D(V_{1})}\sqrt{D(V_{2})}}$$

where:

$$COV\left(V1,V2\right)=\frac{1}{N}\sum_{i=1}^{N}\left(v1_{i}-E\left(V1\right)\right)\left(v2_{i}-E\left(V2\right)\right),$$

$$D\left(V\right)=\frac{1}{N}\sum_{i=1}^{N}\left(v_{i}-E\left(V\right)\right),$$

and

$$E\left(V\right)=\frac{1}{N}\sum_{i=1}^{N}v_{i}.$$

**Figure 20:**
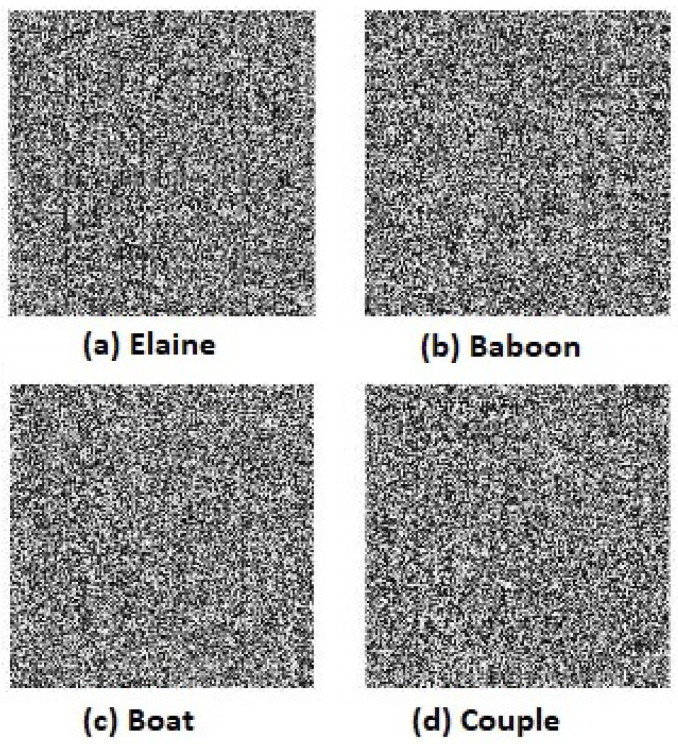
Encrypted images of the proposed algorithm. (**a**) Elaine; (**b**) Baboon; (**c**) Boat; (**d**) Couple.

**Figure 21:**
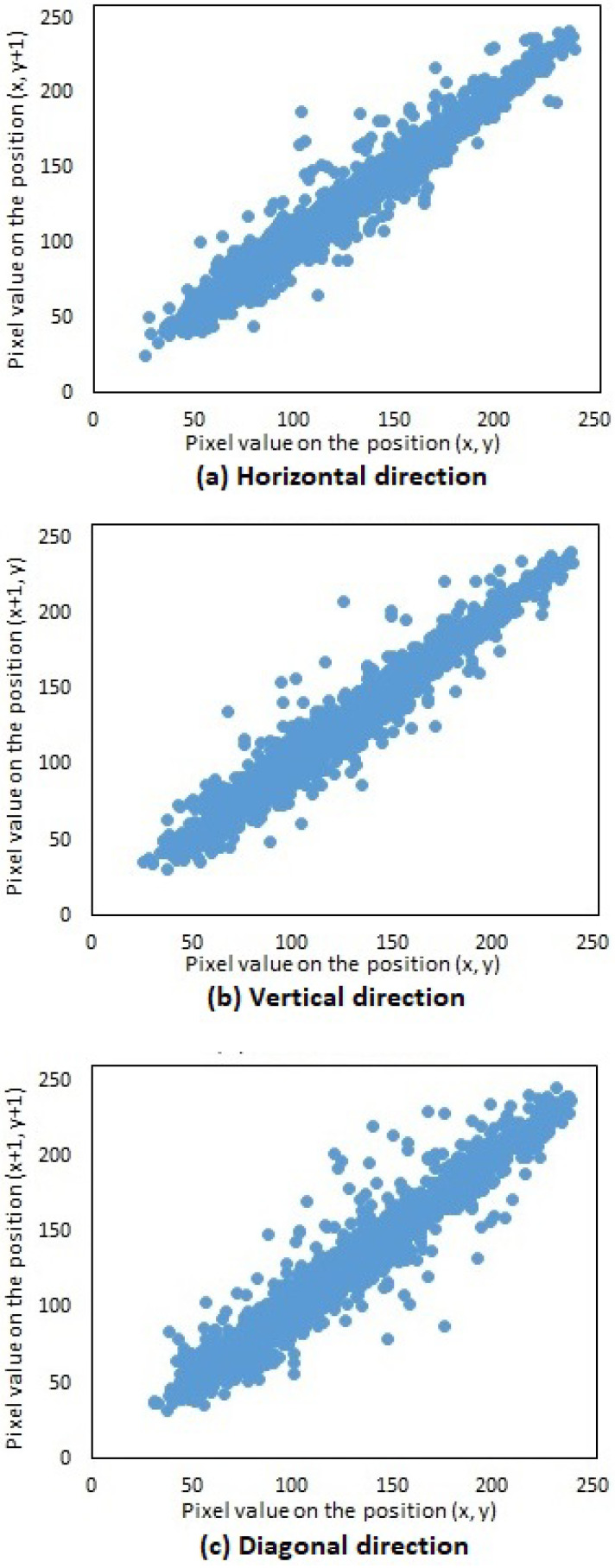
Neighboring pixel correlation of Figure 19a (original image). (**a**) Horizontal direction; (**b**) Vertical direction; (**c**) Diagonal direction.

**Figure 22:**
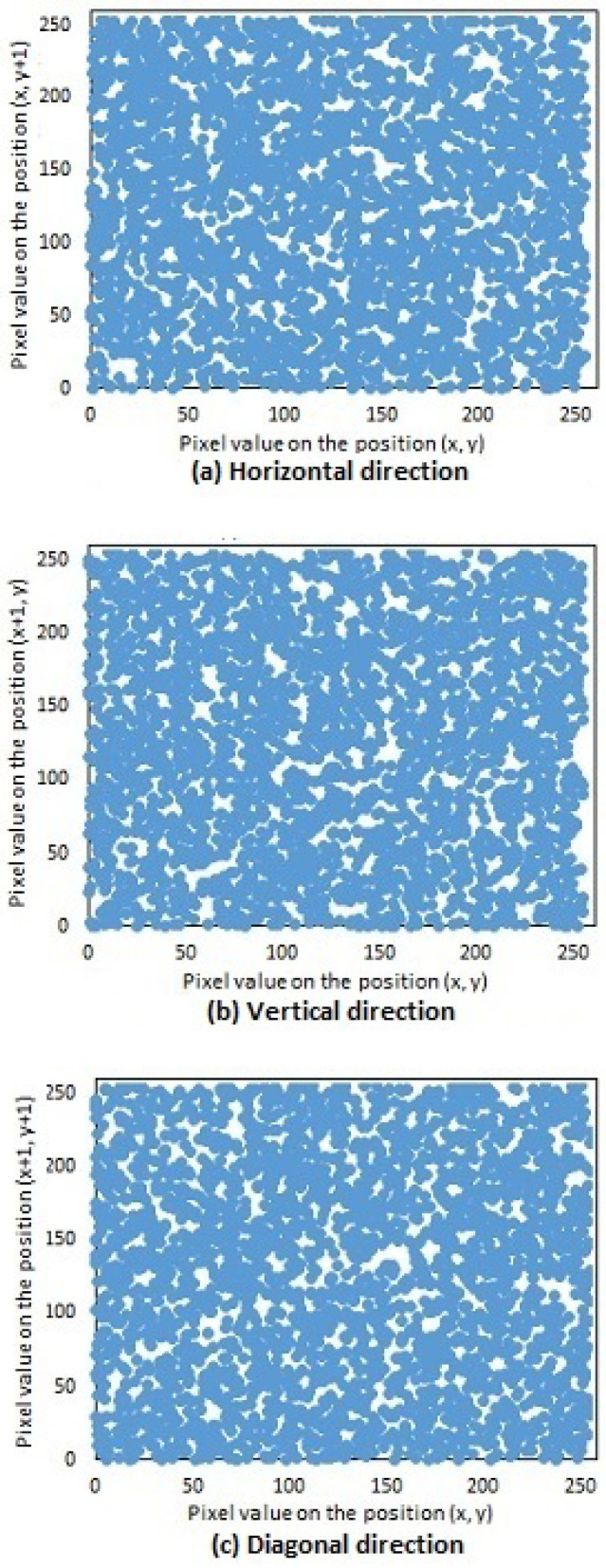
Neighboring pixel correlation of Figure 20a (encrypted image). (**a**) Horizontal direction; (**b**) Vertical direction; (**c**) Diagonal direction.

Three thousand pairs of pixels were selected randomly in all three directions from the two images (original and encrypted); see Figure 19a and Figure 21a, respectively. Then, the correlation coefficients of the two neighboring pixels were computed using Equation (4). The neighboring pixel correlation of Figure 19a and Figure 20a are plotted in Figure 21 and Figure 22. Their correlation coefficients are illustrated in Table 4 and Table 5. The original images’ correlation coefficients were approximately equal to one, while the corresponding ones of encrypted images were approximately equal to zero. The results conclude that the proposed algorithm can conserve the image information.

### 6.7. Analysis of Differential Attack

In the differential attack, the encryption algorithm was used to encrypt the original image before and after modification, then the two encrypted images were compared to discover the link between them [26]. Therefore, a good image encryption algorithm should be the desired property to spread the effect of a minor change in the original image of as much an encrypted image as possible. Number of pixels change rate (NPCR) and unified averaged changed intensity (UACI) are famous measurements, which were used to measure the resistance of the image encryption algorithm for differential attacks. The NPCR and UACI are defined as follows,

(7) $$NPCR=\frac{\sum_{i,j}d\left(i,j\right)}{M\times N}\times 100\%,$$

(8) $$UACI=\frac{1}{M\times N}\left[\sum_{i,j}\frac{|I_{1}\left(i,j\right)-I_{2}\left(i,j\right)|}{255}\right]\times 100\%.$$

where:

(9) $$d\left(i,j\right)=\left\{\begin{array}{cc} 0 & \;\mathrm{if}\;I_{1}\left(i,j\right)=I_{2}\left(i,j\right),\\ 1 & \;\mathrm{if}\;I_{1}\left(i,j\right)\neq I_{2}\left(i,j\right) \end{array}\right.$$

*M* and *N* are the width and height of the original and the encrypted images; $I_{1}$ and $I_{2}$ are the encrypted images before and after one pixel changed from the original image. For example, a pixel position (71,42) was selected randomly, and it has the value 159 in Figure 19a. The pixel value was modified to 244 to examine the ability to combat the differential attacks. Table 6 lists the results of Figure 19a–d. The results show that a small modification in the plain image will result in a big modification in the cipher image. Therefore, the proposed algorithm can face differential attacks.

### 6.8. Chosen/Known Plaintext Attack Analysis

Attackers have used two famous attacks called chosen-plaintext attack and known-plaintext attack for attacking any cryptosystem. The secret keys are not only dependent on the given initial values and system parameters, but also on the plain images. Therefore, when the plain images are changed, the secret keys will be changed in the encryption process. Therefore, attackers cannot take important information by encrypting some predesigned special images. Therefore, the proposed algorithm robustly resisted both attacks.

### 6.9. Noise Attack Analysis

**Figure 23:**
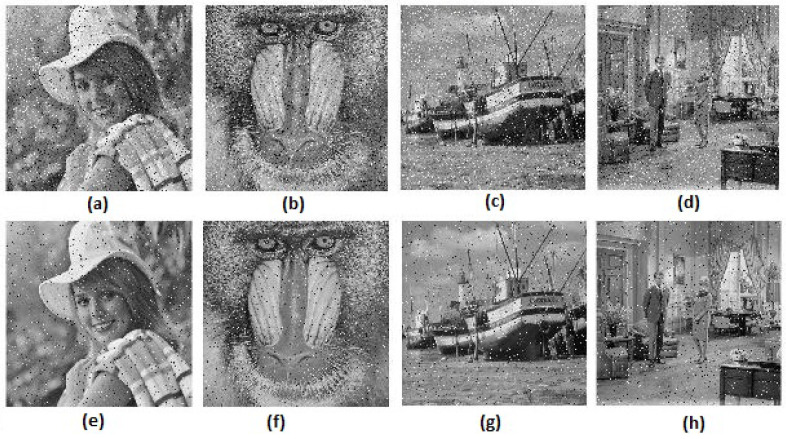
Results of noise attack analysis: (**a**–**d**) the decrypted images after adding Gaussian noise with mean = 0 and variance = 0.001; (**e**–**h**) the decrypted images after added salt and pepper noise with density = 0.05.

The encrypted images in Figure 20 are distorted by adding Gaussian noise with mean = 0 and variance = 0.001 and salt and pepper noise with density = 0.05. The corresponding decrypted images are displayed in Figure 23. Moreover, Table 7 shows the mean squared error (MSE) and the peak signal-to-noise ratio (PSNR) between input images and decrypted images based on the proposed algorithm. Based on Table 7, we can conclude that the proposed algorithm had the highest resisting ability to salt and pepper noise since the PSNR was more than 65 (dB).

### 6.10. Analysis of Occlusion Attack

**Figure 24:**
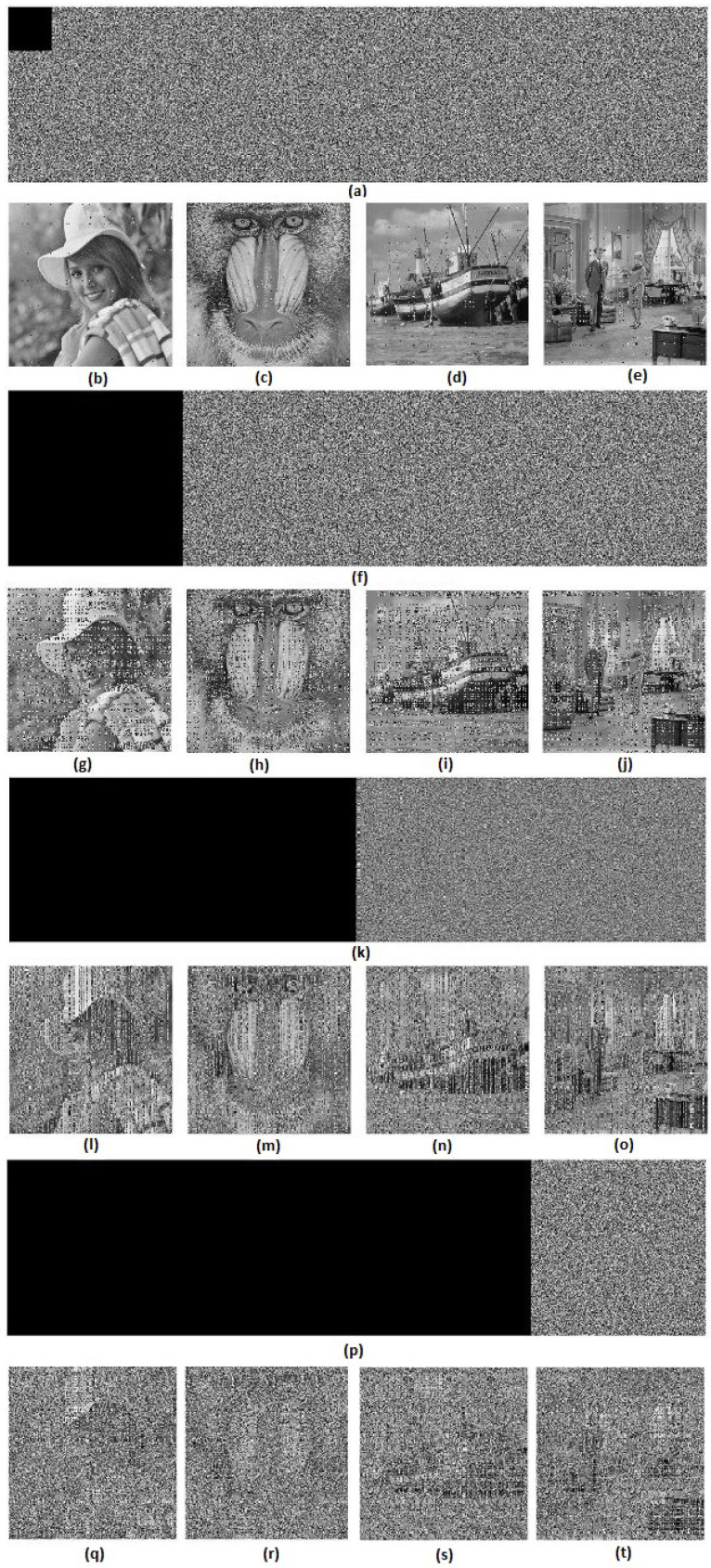
Results of occlusion attack analysis: (**a**,**f**,**k**,**p**) horizontally concatenated encrypted image with a 128×128,512×512,512×1024 and 512×1536 size of occlusion, respectively; (**b**–**e**), (**g**–**j**), (**l**–**o**) and (**q**–**t**) decrypted “Elaine”, “Baboon”, “Boat” and “Couple” images, respectively, when there is a 128×128,512×512,512×1024 and 512×1536 size of occlusion in the horizontally concatenated encrypted image.

The current section is assigned to the analyses of occluded data decryption. Data that are occluded are hidden or ignored data inside the process. Firstly, 128×128,512×512,512×1024 and 512×1536 sized data occlusions of the horizontally concatenated encrypted image were performed. Secondly, the decrypted image of each one was analyzed. Figure 24 shows the results of the occlusion attack. Based on Figure 24, the decrypted images of 128×128,512×512,512×1024 sized occluded encrypted images were disfigured, but discernible by the human eye, while decrypted images of 512×1536 sized occluded encrypted images were not restored. Hence, the proposed algorithm could resist up to a 50% (512×1024) occlusion attack.

## 7. Comparison with Other Algorithms

**Figure 25:**
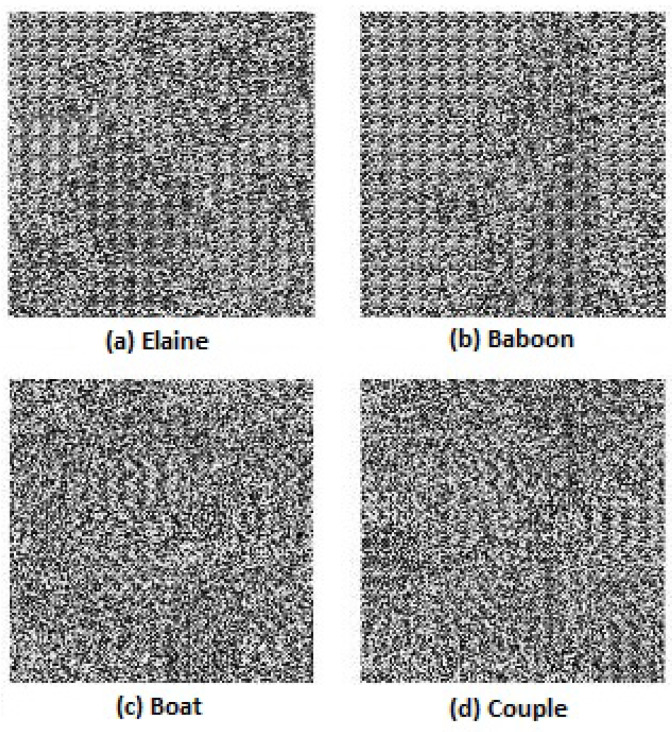
Encrypted images of Zhang’s algorithm.

A comparison between Tang’s algorithm [20] and Zhang’s algorithm was performed in [10]. The result of the comparison concluded that Zhang’s algorithm was faster than Tang’s algorithm. Therefore, a comparison between Zhang’s algorithm and the proposed algorithm is presented. The same four original gray images are chosen as input images and are displayed in Figure 19. Furthermore, the size of MIES = 64×64 is selected. The encrypted images of the proposed algorithm and Zhang’s algorithm are shown in Figure 20 and Figure 25, respectively. The computational times of both algorithms are listed in Table 8. Although the time of Zhang’s algorithm is less than the proposed algorithm, the encrypted images’ histograms of the proposed algorithm are uniformly distributed, and the encrypted images histograms of Zhang’s algorithm are not uniformly distributed (see Figure 13 in [10]). Therefore, the experimental results conclude that the proposed algorithm is efficient. The security of Zhang’s algorithm is a little weaker than the proposed algorithm since the key space size of the proposed algorithm is larger than Zhang’s algorithm and two additional shuffling operations are added to the proposed algorithm, one for PIES and one for the big scrambled image.

## 8. Conclusions

The current paper has proposed a new multiple-image encryption algorithm using combination of MIES and a two-dimensional chaotic economic map. The key space size of the proposed algorithm is $10^{210}$. Therefore, it gives priority to the proposed algorithm to resist against brute-force attack. The experimental results have demonstrated that the proposed algorithm produced encrypted images that have histograms with uniform distributions. In addition, the proposed algorithm has demonstrated that the encrypted images have information entropies close to eight. It robustly resists chosen/known plaintext attacks, has the highest resisting ability to salt and pepper noise and can resist up to a 50% (512×1024) occlusion attack. Comparison experiments with Zhang’s algorithm were performed. Furthermore, the analyses of the algorithm conclude that the proposed algorithm is secure and efficient. It can be applied in several fields like weather forecasting, military, engineering, medicine, science and personal affairs. In this paper, the proposed idea was simulated on grayscale images, which had the same size. In the future, the proposed idea will applied on grayscale images with different sizes.

## Figures and Tables

**Table 1:** Comparison of the current key space with other key spaces in the literature.

Algorithm	Proposed Algorithm	Ref. [10]	Ref. [20]	Ref. [22]
Key Space	$10^{210}$	$10^{60}$	$2^{451}=5.8147\times 10^{135}$	$1.964\times 2^{428}=1.3614\times 10^{129}$

**Table 2:** Comparison of histogram variances between three algorithms.

Algorithm	Tang’s Algorithm [20]	Zhang’s algorithm [10]	Proposed Algorithm
Figure 19a	1261.8	1155.5	1055.5
Figure 19b	1192.3	989.6	984.8
Figure 19c	1213.1	1111.6	1079.7
Figure 19d	8710.3	929.6	916.9

**Table 3:** Information entropy for the encrypted images in Figure 14.

Images	(a)	(b)	(c)
Entropy	7.9984	7.9987	7.9986
Images	(d)	(e)	(f)
Entropy	7.9982	7.9986	7.9983
Images	(g)	(h)	(i)
Entropy	7.9986	7.9989	7.9986

**Table 4:** The original images’ correlation.

Directions	Horizontal	Vertical	Diagonal
Figure 19a	0.9757	0.9729	0.9685
Figure 19b	0.9228	0.8597	0.8476
Figure 19c	0.9383	0.9715	0.9224
Figure 19d	0.9439	0.8687	0.8334

**Table 5:** The encrypted images’ correlations.

Directions	Horizontal	Vertical	Diagonal
Figure 20a	−0.0035	0.0014	0.0007
Figure 20b	0.0036	−0.0005	0.0010
Figure 20c	0.0015	0.0013	−0.0017
Figure 20d	−0.0008	0.0008	0.0031

**Table 6:** The values of number of pixels change rate (NPCR) and unified averaged changed intensity (UACI) for Figure 19.

Image	NPCR	UACI
Figure 19a	99.62%	33.44%
Figure 19b	99.61%	33.85%
Figure 19c	99.62%	33.42%
Figure 19d	99.60%	33.18%

**Table 7:** Measurements of the noise attacks of the proposed algorithm.

Image	Noise	MSE	PSNR
Figure 23a		0.0603	60.3255
Figure 23b	Gaussian	0.0602	60.3346
Figure 23c	variance = 0.001	0.0474	61.3691
Figure 23d		0.0560	60.6455
Figure 23a		0.0184	65.4921
Figure 23b	salt & pepper	0.0162	66.0291
Figure 23c	density = 0.05	0.0172	65.7719
Figure 23d		0.0155	66.2276

**Table 8:** Computational time (seconds).

Algorithm	Time
Zhang’s algorithm [10]	2.169
Proposed algorithm	2.386
